# Letter to the Editor: Closing the loop in radiology—patient access to reports as an additional safety layer

**DOI:** 10.1186/s13244-026-02412-3

**Published:** 2026-09-22

**Authors:** Yoshito Tsushima, Ayako Taketomi-Takahashi

**Affiliations:** Department of Diagnostic Radiology and Nuclear Medicine, Gunma University Graduate School of Medicine, Maebashi, Japan

Patient access to electronic health records, including radiology reports, has expanded rapidly through patient portals. Although this trend has mainly been discussed in terms of transparency, patient engagement, and shared decision-making, its implications for diagnostic safety in radiology have received comparatively little attention.

OpenNotes has shown that patients value access to their clinical records and become more engaged in their care when able to review physician documentation [1, 2]. Several countries have implemented nationwide systems that provide electronic access to medical records, including imaging reports. In Finland, integration of radiology information systems and PACS into the national Kanta system enables patients to access their records electronically [3]. In the United States, the 21st Century Cures Act requires timely patient access to electronic health information, including radiology reports [4]. Implementation remains heterogeneous, with legal requirements established in only 29 of 53 WHO European Region member states [5]. This variability provides an opportunity to integrate patient access into diagnostic safety strategies rather than viewing it solely as a transparency initiative.

Radiology provides an ideal setting to examine the safety implications of patient access because communication failures related to imaging findings remain a major source of diagnostic delay and error. Clinically significant findings may not always be recognized or acted upon, particularly when multiple providers are involved. Reliance on written reports alone increases the risk of missed or delayed follow-up.

Among these challenges, incidental findings represent a well-recognized and clinically important example. Incidental findings are abnormalities unrelated to the original imaging indication. Although many are benign, some require follow-up imaging or further evaluation. Because management is often deferred rather than immediate, these findings are particularly vulnerable to communication failures. Prior studies have reported that 20–40% of recommended follow-up imaging examinations are not completed [6], highlighting persistent gaps between detection and action.

These communication failures have led to the concept of “closing the loop”, ensuring that important imaging findings are identified, communicated, and acted upon [7]. Proposed strategies include structured reporting, automated tracking systems, incidental finding programs, and systems to identify unread radiology reports [8, 9]. These approaches primarily strengthen communication and accountability among healthcare professionals.

In this context, patient access to radiology reports may represent an additional, and currently underrecognized, layer within the loop-closure process. Although direct evidence specifically linking patient access to radiology reports with improved follow-up rates or clinical outcomes remains limited, empirical studies of OpenNotes and electronic health record access have demonstrated that patients actively engage with their medical records, better understand their care, and can identify documentation errors that might otherwise go unnoticed. These findings support the concept that patient access may serve as an additional safeguard in the diagnostic process and should be further evaluated as a component of loop-closure strategies [10–12]. When patients can directly review their imaging reports, they may become aware of findings that require follow-up, providing an additional opportunity to recognize overlooked findings and facilitate timely follow-up. This may be particularly relevant in situations where communication between clinicians is incomplete or when patients transition between providers. In such cases, patient awareness could serve as a supplementary safeguard against missed follow-up.

Importantly, this perspective does not shift responsibility for follow-up onto patients. Radiologists, referring clinicians, and healthcare systems remain primarily responsible for appropriate communication and management. Rather, patient access should be viewed as an additional layer of redundancy that complements, but does not replace, existing safety mechanisms and may enhance system resilience.

The potential benefits of patient access are also likely to vary across patient populations and healthcare systems. Factors such as health literacy, digital literacy, language proficiency, and socioeconomic circumstances may influence patients’ ability to understand and act upon radiology reports. Likewise, implementation strategies should be adapted to local healthcare systems, cultural contexts, and available resources rather than adopting a uniform approach.

Integrating patients into the communication loop also raises implementation challenges. Radiology reports often contain technical language and uncertain findings. Immediate access without explanation may cause confusion or distress. Although these concerns are widely discussed, evidence suggests that patients can engage constructively with medical information when appropriate support is available [2]. Some concerns may also reflect a tendency to overestimate negative reactions, a phenomenon described as “elite panic” [13].

Balancing transparency and safety requires thoughtful system design, including appropriate timing of report release, patient-friendly language, and interpretive support. Without such measures, increased access could contribute to anxiety or inappropriate healthcare use. Conversely, well-designed systems may enable patients to participate in recognizing clinically important findings. Practical implementation may include patient-friendly report summaries, standardized highlighting of recommended follow-up, and educational resources integrated into patient portals. Such measures could facilitate patient participation while preserving clinicians’ primary responsibility. Our institutional experience indicates that patients do review their radiology reports and may raise questions regarding recommended follow-up. Although anecdotal, this experience suggests that patient access can function as an additional communication pathway within loop-closure strategies.

Patient access to medical information will likely continue to expand and should be viewed not only as a transparency initiative but also as an opportunity to strengthen diagnostic safety. Radiology should deliberately integrate patient access into loop-closure strategies through structured patient-facing communication while maintaining professional responsibility for follow-up. Professional societies can help establish guidance, and future research should determine whether this approach improves follow-up and patient outcomes. Recognizing patients as active participants may ultimately contribute to more resilient diagnostic systems.
